# SNAT1 (SLC38A1) Is Not the Main Glutamine Transporter in Melanoma, but Controls Metabolism via Glutamine-Dependent Activation of P62 (SQSTM1)/cMYC-Axis

**DOI:** 10.3390/cancers18071068

**Published:** 2026-03-25

**Authors:** Sandra Lörentz, Ines Böhme-Schäfer, Jörg König, Heinrich Sticht, Anja Katrin Bosserhoff

**Affiliations:** 1 Department of Biochemistry and Molecular Medicine, Institute of Biochemistry, Friedrich-Alexander-Universität Erlangen-Nürnberg (FAU), Fahrstraße 17, 91054 Erlangen, Germany; sandra.loerentz@fau.de (S.L.); ines.boehme@fau.de (I.B.-S.); 2 Department Hamm 2, Hochschule Hamm Lippstadt, Marker Allee 76–78, 59063 Hamm, Germany; 3 Department of Clinical Pharmacology and Toxicology, Institute of Experimental and Clinical Pharmacology and Toxicology, Friedrich-Alexander-Universität Erlangen-Nürnberg (FAU), Fahrstraße 17, 91054 Erlangen, Germany; joerg.koenig@fau.de; 4 FAU NeW Research Center New Bioactive Compounds, Friedrich-Alexander-Universität Erlangen-Nürnberg (FAU), 91054 Erlangen, Germany; 5 Division of Bioinformatics, Institute of Biochemistry, Friedrich-Alexander-Universität Erlangen-Nürnberg (FAU), Fahrstraße 17, 91054 Erlangen, Germany; heinrich.sticht@fau.de; 6 Bavarian Cancer Research Center (BZKF), 91054 Erlangen, Germany; 7 CCC Erlangen-EMN: Comprehensive Cancer Center Erlangen-EMN, 91054 Erlangen, Germany; 8 CCC WERA: Comprehensive Cancer Center Alliance WERA, 91054 Erlangen, Germany

**Keywords:** melanoma, glutamine transport, tumor metabolism, SNAT1 (SLC38A1), transceptor

## Abstract

**Background**: Tumor cells can reprogram their metabolism, constituting a hallmark of cancer that plays a crucial role in tumor progression. As tumor cells exhibit an increased demand for nutrients, e.g., amino acids, they rely on extracellular sources and show deregulation of transport proteins. Among these, SNAT1 (SLC38A1) is described as the loader for glutamine that is responsible for the main influx of this amino acid. The aim of this study was to assess the molecular function of SNAT1 in melanoma regarding its role in amino acid transport and regulation of cellular metabolism. **Methods**: siPool-mediated downregulation of SNAT1 expression in melanoma cell lines was used to investigate the molecular function of this protein. Glutamine transport was assessed by measuring the intracellular and extracellular concentrations of glutamine. Regulation of downstream effectors was evaluated with qRT-PCR and Western Blot. Metabolism was investigated by performing Seahorse flux analysis. Mitochondrial staining was examined via flow cytometry. Protein interaction was assessed with Co-IP, and in silico modeling of protein interaction was performed with AlphaFold3. **Results**: In this study, we uncovered the new finding that SNAT1 is not primarily implicated in glutamine influx into melanoma cells but in signaling in response to extracellular glutamine. We identified P62 and cMYC as downstream effectors of SNAT1. By activating the P62/cMYC-axis and target genes of cMYC, SNAT1 modulates the metabolism of melanoma cells depending on the glutamine level. SNAT1 and P62 are interaction partners. **Conclusions**: This finding newly suggests that SNAT1 may function as a sensor or receptor (“transceptor”) for glutamine rather than being a direct and primary glutamine transporter, and could open up new therapeutic options targeting melanoma cells.

## Simple Summary

Malignant melanoma is the deadliest form of skin cancer and originates from melanocytes. Cancer cells can reprogram their metabolism, which contributes to tumor progression. Tumor cells exhibit an adapted expression of transport proteins to cover their increased demand for nutrients. Cancer cells are characterized by an elevated consumption rate of the amino acid glutamine, which is also known as glutamine addiction. In the literature, SNAT1 (*SLC38A1*) is described as the main transporter for glutamine uptake. The aim of this study was to investigate the molecular function of SNAT1 in human melanoma in more detail. We revealed that SNAT1 is not the main glutamine transporter in melanoma. Instead, it activates the P62/cMYC-axis depending on the extracellular glutamine level. This glutamine-dependent transceptor modulates the metabolism of melanoma cells. Therefore, SNAT1 constitutes a promising target protein for the development of new therapeutic options for melanoma patients.

## 1. Introduction

The incidence of malignant melanoma has been rising for decades. Melanoma is a highly metastatic and very aggressive cancer type, being the most lethal form of skin cancer [1]. Patients diagnosed with stage IV melanoma exhibit a 5-year survival rate of only 32% despite undergoing treatment [2]. The emergence of treatment resistance highlights the great need for research on melanoma development and progression, which will enable the development of new treatment approaches [3].

In general, tumor cells exhibit an increased proliferation rate compared to healthy cells. To meet their elevated need for nutrients and ATP to support continuous proliferation, tumor cells reprogram their metabolism. This represents one of the new hallmarks of cancer [4]. Nutrient transporters, which are located upstream of metabolic pathways relevant for tumor cells, have only been gaining more attention in recent years. Tumor cells adapt their substrate fluxes to meet the demands of proliferation, which is connected to the deregulation of numerous transport proteins. Their elevated consumption of amino acids renders tumor cells dependent on upregulation of the corresponding amino acid transporters [5]. Melanoma cells express a defined set of glutamine influx transporters, including ASCT2 (*SLC1A5*), LAT1 (*SLC3A2/SLC7A5*), y+LAT1 (*SLC3A2/SLC7A7*), LAT2 (*SLC3A2/SLC7A8*), y+LAT2 (*SLC3A2/SLC7A6*), SNAT1 (*SLC38A1*), SNAT2 (*SLC38A2*), SNAT4 (*SLC38A4*) [6,7], SNAT5 (*SLC38A5*), and SNAT7 (*SLC38A7*) (proteinatlas.org).

SNAT1 belongs to the system A amino acid transporters, as well as SNAT2 and SNAT4. By mediating the influx of sodium ions and neutral amino acids, SNAT1 is known to be a secondary active transporter [8,9]. Transporters of the solute carrier family 38 (SLC38) are described as the main glutamine transporters, especially SNAT1, being responsible for the main glutamine uptake for glutaminolysis [10]. Some studies found that SNAT1 is involved in activation of the protein kinase B (AKT)/mTOR signaling pathway [11,12,13], which is frequently activated in melanoma [14]. SNAT1 protein expression is increased in human cancer tissue and cell lines of some tumor entities [11,12,15], including melanoma [7]. Elevated expression level of SNAT1 is associated with shorter median survival of patients with different cancer types [11,12,15,16], highlighting that SNAT1 constitutes an unfavorable prognostic marker for several tumor entities.

In a previous study, we showed that SNAT1 expression in melanoma is elevated in vitro and in vivo compared to melanocytes and healthy tissue [7]. Moreover, siPool-mediated downregulation of SNAT1 expression and application of MeAIB, a competitive inhibitor for system A amino acid transporters, leads to reduced proliferation, viability, colony formation, migration, and invasion, while simultaneously inducing senescence in melanoma cells [7]. However, the molecular function of SNAT1 in melanoma and its implication in amino acid transport and metabolism remain to be elucidated in more detail.

In this study, we revealed that SNAT1 is localized in the Golgi and plasma membrane of human melanoma cells. Interestingly and newly, we discovered that in melanoma SNAT1 does not primarily function as a glutamine influx transporter. Instead, SNAT1 activates the P62/cMYC-axis in a glutamine-dependent manner by releasing captured P62, thereby regulating cellular metabolism.

## 2. Materials and Methods

### 2.1. Culturing of Cells

The human melanoma cell line Mel Juso (RRID:CVCL_1403, primary cutaneous melanoma, mutation: NRASQ61L) was cultivated in RPMI-1640 medium (Roswell Park Memorial Institute, Buffalo, NY, USA) and human melanoma cell line Mel Im (RRID: CVCL_3980, cutaneous melanoma metastasis, mutation: BRAFV600E) was cultured in DMEM D6046 low glucose (Dulbecco’s Modified Eagle’s Medium, Sigma Life Science, St. Louis, MO, USA). Both melanoma cell lines were provided by Judith Johnson (LMU, München, Germany). Both media were supplemented with 10% fetal calf serum (Sigma-Aldrich, München, Germany), penicillin (400 U/mL) and streptomycin (50 µg/mL). RPMI-1640 medium was also supplemented with NaHCO_3_ (Sigma-Aldrich). Human melanoma cells were cultivated in a humidified atmosphere at a temperature of 37 °C, containing 5% CO_2_ (Mel Juso) or 8% CO_2_ (Mel Im). The two cell lines HEK Ctr (ATCC, Manassas, VA, USA) and HEK-SNAT1 were cultivated in DMEM D6046 low glucose medium that was supplemented with 10% fetal calf serum, geneticin/G418 (800 µg/mL), penicillin (400 U/mL), and streptomycin (50 µg/mL). Both HEK cell lines were incubated in a humidified atmosphere containing 5% CO_2_ at a temperature of 37 °C.

### 2.2. Transfection with siRNA and Plasmid

Cells were transiently transfected with siRNA using the Lipofectamine RNAiMAX reagent (Invitrogen, ThermoFisher Scientific, Darmstadt, Germany) in combination with a siPool targeting SNAT1 that consists of 30 specific siRNAs (functionally verified by siTOOLs Biotech, Planegg, Germany) or a negative control siPool, respectively. The experimental setup of seeding of the melanoma cell lines Mel Im and Mel Juso and their transfection for 96 h was performed as described in a previous publication [7]. The efficiency of SNAT1 mRNA and protein expression downregulation has been described in a previous publication, showing that after a transfection period of 96 h, protein expression of SNAT1 is reduced to 28% in Mel Im and to 60% in Mel Juso cells [7]. Knockdown efficiency of SNAT1 was controlled carefully on a regular basis. All of the following functional experiments were conducted after a total transfection period of 96 h.

Transfection with ZsGreen1-cMYC/pLVX-Puromycin vector was performed using 0.5 µg plasmid DNA and Lipofectamine LTX Plus reagent (Invitrogen, ThermoFisher Scientific) for a transfection period of 48 h. Andre Catic kindly gifted the ZsGreen1-cMYC/pLVX-Puromycin vector (RRID:Addgene_180278). pLenti-C-GFP (OriGene, Rockville, MD, USA; RRID:Addgene_17448) served as a control plasmid in our experiments.

### 2.3. Cloning of Human SLC38A1 cDNA and Establishment of HEK-SNAT1 Cells

The SLC38A1 cDNA encoding human SNAT1 was cloned by using reverse transcription-PCR (RT-PCR). As primers, hSLC38A1forHindIII (GACAAGCTTATGATGCATTTCAAAAGTGGACTCG) and hSLC38A1revBamHI (GACGGATCCGCCTAAGGAGGTTGTACCTGG) were chosen. Total RNA was isolated from Mel Juso cells and served as a template. The cDNA generated from total RNA was cloned into a pCR2.1.TOPO vector (Thermo Fisher Scientific, Bonn, Germany) and subcloned into a pcDNA3.1(+) expression vector (Thermo Fisher Scientific; RRID:Addgene_100544). Base pair exchanges that lead to amino acid substitutions after translation were corrected via site-directed mutagenesis. The generated plasmid pSNAT1.31 contains a cDNA, which encodes the human SNAT1 protein that is 100% identical to the reference sequence (NM_030674.3). Human HEK293 cells (RRID CVCL_0045; ATCC) were transfected with pSNAT1.31 using the Effectene transfection reagent (Qiagen, Hilden, Germany). Cell clones that exhibit a stable expression of the human SLC38A1 mRNA were selected by quantitative real-time PCR (qRT-PCR). The cell clone with the highest mRNA expression of SLC38A1 in relation to β-actin (HEK-SNAT1: 124.7%) was used for subsequent experiments.

### 2.4. mRNA Expression Analysis

Total RNA Kit I (Omega Bio-Tek, Norcross, GA, USA) was used for the isolation of total cellular RNA from melanoma cells according to the manufacturer’s instructions. In total, 500 ng mRNA were utilized for reverse transcription into cDNA by using the SuperScript II Reverse Transcriptase Kit (Invitrogen, ThermoFisher Scientific). For the investigation of mRNA expression level, we conducted qRT-PCR using the LightCycler480 II (Roche, Mannheim, Germany) (see Table 1 for primer sequences). Target gene expression was normalized to β-actin for analysis.

### 2.5. miRNA Expression Analysis

To isolate total cellular micro-RNA (miRNA), the miRNeasy Tissue/Cells Advanced Mini Kit (Qiagen) was used following the manufacturer’s instructions. In total, 500 ng of miRNA were utilized for reverse transcription into miRNA-cDNA (miRcDNA). For this purpose, the miRCURY LNA™ RT kit (Qiagen) was used following the manufacturer’s protocol. For expression analysis of miR-let7a, we performed qRT-PCR on a LightCycler480 II device (Roche) with the miRCURY LNA SYBR Green PCR kit (Qiagen). The hsa-miR-let7a-5p miRCURY LNA miRNA PCR assay (Qiagen) and the U6 SNRA miRCURY LNA miRNA PCR assay (Qiagen) were used for primers. The expression of miR-let7a was normalized to the expression level of U6.

### 2.6. Protein Expression Analysis

To isolate protein and subsequently analyze protein expression, the cells were lysed using a radioimmunoprecipitation assay buffer (50 mM Tris/HCl, pH 7.4, 150 mM NaCl, 1% NP40, 0.5% sodium deoxycholate, 0.1% SDS, protease inhibitor). Each lane was loaded with 20 µg protein. Proteins were separated via SDS-PAGE using polyacrylamide gels. Afterwards, proteins were blotted onto a polyvinylidene fluoride membrane (Bio-Rad Laboratories Inc., Hercules, CA, USA). Membranes were blocked with 5% bovine serum albumin (BSA)/TBS-T, 5% non-fat dried milk (MP)/TBS-T or 3% BSA + 5% MP. The following primary antibodies were incubated overnight at 4 °C: SNAT1 (1:1000 in 5% BSA, Cell Signaling Technology, Danvers, MA, USA; Cat# 36057, RRID:AB_2799092), P62 (1:1000 in 3% BSA + 5% MP, Santa Cruz, Dallas, TX, USA; Cat# sc-28359, RRID:AB_628279), LC3-II (1:1000 in 5% BSA, Proteintech ChromoTek GmbH, Planegg-Martinsried, Germany; Cat# 81004-1-RR, RRID:AB_2923695), p-mTOR (1:1000 in 5% BSA, Cell Signaling; Cat# 2971, RRID:AB_330970), p-AKT (1:3000 in 5% BSA, Cell Signaling; Cat# 4060, RRID:AB_2315049), AKT (1:2000 in 5% BSA, Cell Signaling; Cat# 9272, RRID:AB_329827), p-ERK (1:3000 in 5% BSA, Cell Signaling; Cat# 4370, RRID:AB_2315112), ERK (1:1000 in 5% BSA, Cell Signaling; Cat# 9102, RRID:AB_330744), and cMYC (1:1000 in 5% MP, Cell Signaling; Cat# 9402, RRID:AB_2151827). For loading control, primary antibody against β-actin (1:5000 in TBS-T, Sigma-Aldrich; Cat# A5441, RRID:AB_476744) or vinculin (1:1000 in 5% MP, Santa Cruz; Cat# sc-73614, RRID:AB_1131294) was applied. Secondary antibodies were conjugated to horseradish peroxidase (Cell Signaling; Cat# 7074, RRID:AB_2099233; Cat# 7076, RRID:AB_330924). The Clarity™ Western ECL Substrate kit (Bio-Rad Laboratories Inc.) was used for visualization. To detect the signal, the Chemostar chemiluminescence imager (Intas Science Imaging Instruments GmbH, Göttingen, Germany) was utilized. Densitometric analysis was performed using the LabImage software (V 4.2.3; Kapelan Bio-Imaging GmbH, Leipzig, Germany).

The Protein Deglycosylation Mix II (New England Biolabs GmbH, Frankfurt am Main, Germany) was used for the deglycosylation of proteins, following the manufacturer’s instructions. In total, 60 µg of protein were loaded per lane and separated via SDS-PAGE on polyacrylamide gels. The following Western Blot analysis was performed as described above.

### 2.7. Co-Immunoprecipitation

Pierce™ protein A/G magnetic beads (Thermo Fisher Scientific) were used to carry out co-immunoprecipitations (Co-IP). For this purpose, pre-cleared protein lysate (200 µg) was incubated overnight at 4 °C with SNAT1 antibody (Cell Signaling, 1:50; Cat# 36057, RRID:AB_2799092) or with rabbit mAb IgG XP^®^ isotype control (Cell Signaling, 1:50; Cat# 3900, RRID:AB_1550038). Washed magnetic beads were added the following day and incubated again overnight at 4 °C. Proteins that had bound to the magnetic beads were eluted by incubation for 5 min at 90 °C. Subsequently, eluted proteins were loaded onto a polyacrylamide gel for SDS-PAGE. For Western Blot analysis, see explanation above. The Western Blots were incubated with P62 (Santa Cruz) and SNAT1 antibody (Cell Signaling) overnight at 4 °C. VeriBlot for IP Detection Reagent coupled to horseradish peroxidase (abcam, Cambridge, UK) was used as secondary antibody. In total, 20 µg of protein were used as the input control.

### 2.8. Immunofluorescence Staining

To determine the intracellular localization of SNAT1, we seeded 10,000 untreated melanoma cells onto cover slips (18 mm; Carl-Roth, Karlsruhe, Germany) in 12-well culture plates (Corning Incorporated, Corning, Wiesbaden, Germany) and incubated them overnight. The next day, the cells were fixed with 4% paraformaldehyde/PBS for 15 min. To block and permeabilize the cells, the coverslips were incubated with 1% BSA + 0.1% Triton X-100/PBS for 30 min. Primary antibody for SNAT1 (1:1000, rabbit, Sigma-Aldrich; Cat# HPA052272, RRID:AB_2681781) was applied and incubated overnight at 4 °C. The following day, secondary AlexaFluor488 antibody (1:2000 in 1% BSA/PBS, goat, ThermoFisher Scientific; Cat# A-11034, RRID:AB_2576217) was applied and incubated for 1 h. Afterwards, the coverslips were incubated with primary anti-Golgi Protein 58K antibody (1:200 in 1% BSA/PBS, mouse, abcam; Cat# ab27043, RRID:AB_2107005) overnight at 4 °C and subsequently with secondary AlexaFluorPlus555 antibody (1:2000 in 1% BSA/PBS, goat, ThermoFisher Scientific; Cat# A32727, RRID:AB_2633276) for 1 h. Nuclei were stained by application of 4′,6-diamidino-2-phenylindole (DAPI) (1:10,000 in 1% BSA/PBS, Merck KGaA, Darmstadt, Germany) for 30 min. Aqua Poly/Mount (Polysciences Inc., Warrington, PA, USA) was used for embedding the coverslips. Microscope slides were stored in the dark at a temperature of 4 °C. The stained cells were imaged and analyzed using an Olympus IX83P2ZF microscope and Olympus CellSens Dimension software (version 2.3; Olympus, Hamburg, Germany).

### 2.9. Surface Biotinylation of Plasma Membrane Proteins

Two million cells were washed three times with ice-cold PBS and resuspended in 1 mL PBS. After adding biotin reagent solution (200 µL, 10 mM; EZ-Link™ Sulfo-NHS-LC-Biotin, Thermo Fisher Scientific), resulting in a 2 mM biotin-cell mixture, the cells were incubated at low speed on an overhead shaker for 30 min. Subsequently, the cells were washed three times with 100 mM glycine/PBS and lysed as described above. Biotinylated proteins were enriched and purified by using Streptavidin Mag Sepharose™ beads (Sigma-Aldrich) according to the manufacturer’s instructions. For the subsequent protein expression analysis, see the explanation above.

### 2.10. Measurement of Glutamine Uptake

L-glutamine uptake of melanoma cells was measured using a standardized transport assay. In total, 400,000 Mel Im and 500,000 Mel Juso that were previously transfected transiently with an siPool (please see Section 2.2 for details) were seeded per well of a six-well plate. Adherent cells were incubated overnight with glutamine-free medium to starve the cells for glutamine. The next day, the melanoma cells were washed twice with prewarmed uptake buffer (142 mM NaCl, 12.5 mM HEPES, 5 mM glucose, 5 mM KCl, 1.5 mM CaCl_2_, 1.2 mM MgSO_4_, 1 mM K_2_HPO_4_; pH 7.3). Afterwards, the cells were incubated with 800 µL uptake buffer (100 µM unlabeled and [3H]-labeled L-glutamine) for 5 min. Inhibition experiments were conducted by adding 10 mM MeAIB, 20 mM MeAIB or 10 mM unlabeled L-glutamine at a temperature of 37 °C. To investigate whether L-glutamine uptake into melanoma cells is transporter-mediated, the last step was performed at 4 °C. Following this incubation step, the melanoma cells were washed three times with ice-cold uptake buffer and lyzed with 0.2% SDS. The amount of intracellular radioactivity was determined with the liquid scintillation counter TriCarb 2800 (Perkin Elmer Life and Analytical Sciences GmbH, Rodgau, Germany). The protein concentration of each sample was assessed with a bicinchoninic acid assay (BCA Protein Assay Kit, Thermo Fisher Scientific). Uptake of [3H]-labeled L-glutamine was measured as counts per minute and was normalized to both protein concentration and to its respective control cells.

### 2.11. Extracellular Glutamine Measurement

To functionally inhibit SNAT1, melanoma cells were incubated with the amino acid analog α-(methylamino)isobutyric acid (MeAIB) (Sigma-Aldrich) at 20 mM for 2 h. V-9302 (MedChemExpress, Monmouth Junction, NJ, USA), which is an ASCT2 inhibitor, was used at 25 µM for 2 h. Control cells (Ctr) were treated with an equal amount of the respective solvent (ddH_2_O or DMSO). Extracellular glutamine concentration was determined by using the Glutamine/Glutamate-Glo™ Assay (Promega GmbH, Walldorf, Germany) in accordance with the manufacturer’s instructions.

### 2.12. H_2_DCFDA Staining

To assess the formation of ROS, melanoma cell lines Mel Im and Mel Juso were washed with PBS and subsequently incubated with 10 µM of 2′,7′-dichlorodihydrofluorescein diacetate (H_2_DCFDA) (Sigma-Aldrich) in PBS for 15 min at 37 °C. Afterwards, the cells were washed with 10% fetal calf serum/PBS and with PBS and subsequently trypsinized and centrifuged. After washing the cells with PBS, they were centrifuged at 4 °C and resuspended in 2% BSA/PBS. The cells’ fluorescence intensity was assessed via flow cytometry using the BD LSRFortessa™ X-20 instrument (Becton Dickinson, Franklin Lakes, NJ, USA). Flow cytometric data were analyzed with the BD FACSDiva™ software (V 8.0; Becton Dickinson). As a positive control, melanoma cells were treated with cold atmospheric plasma by applying the plasma care^®^ device (terraplasma medical GmbH, Garching, Germany) for 2 min in accordance with the manufacturer’s protocol.

### 2.13. Sequencing

Microsynth Seqlab GmbH (Göttingen, Germany) conducted Sanger sequencing of SLC38A1 and used the following primers: exon3fwd (GGTGTCAAGCTTCCATTTTGGTA), exon9fwd (TTCTCCCTCTGTGTCTCTTGA), exon10rev (CCATACAGCTCAAGGAAAATCCAC), and exon17rev (TGGTGAGAGATTGCTGATGTGT).

### 2.14. Seahorse Extracellular Flux Analyses

For Seahorse extracellular flux analyses, 20,000 Mel Im and 30,000 Mel Juso were seeded per well into XFp microplates (Agilent Technologies Inc., Santa Clara, CA, USA) and incubated overnight. A Seahorse XFp sensor cartridge (Agilent Technologies Inc.) was hydrated with water and incubated overnight, as well. The Seahorse XFp sensor cartridge was hydrated with calibrant solution (Agilent Technologies Inc.) the next day. ATP Rate Assay and Mito Stress Test were performed in accordance with the manufacturer’s protocols using the Seahorse XFp Analyzer (Agilent Technologies Inc.). For the ATP Rate Assay, Oligomycin (1.5 µM) and Rotenone/Antimycin A (0.5 µM) were applied sequentially to the cells. For the Mito Stress Test, Oligomycin (1.5 µM), carbonyl cyanide-p-trifluoromethoxyphenylhydrazone (FCCP) (1.5 µM) and Rotenone/Antimycin A (0.5 µM) were applied sequentially to the melanoma cells. Data analysis was performed with the Seahorse Analytics software (version 1.0.0-712; Agilent Technologies Inc.). To account for differences in proliferation, the measured raw data of oxygen consumption rate (OCR) and extracellular acidification rate (ECAR) were normalized to cell number. This was determined by analyzing bright field microscopy images acquired with an Olympus IX83P2ZF microscope (Olympus) and counting cells by using the Cell Counter PlugIn of the Image J software (version 1.48; NIH, USA; RRID:SCR_003070).

### 2.15. Glucose Measurement

To determine glucose consumption of the cells, the cells’ medium was changed, and supernatant was collected subsequently at the indicated time points after medium change. For normalization, cells were harvested and counted at each time point. Glucose concentration of supernatants was determined using the FreeStyle Precision Neo (Abbott Diabetes Care Ltd., Wiesbaden, Germany) and corresponding FreeStyle Precision blood glucose test strips (Abbott Diabetes Care Ltd.).

### 2.16. Mitochondrial Analysis

For analysis of mitochondrial integrity, 200,000 melanoma cells were stained with Mito Tracker™ DeepRed (Invitrogen, ThermoFisher Scientific) (62.5 pM) and tetramethylrhodamin-methylester (TMRM) (1 pM) (Invitrogen, ThermoFisher Scientific) for 30 min at 37 °C. For each sample, 10,000 cells were measured via flow cytometry using a BD LSRFortessa™ X-20 cytometer (Becton Dickinson). For data analysis, BD FACSDiva™ (V 8.0; Becton Dickinson) software was used. FCCP (1 µM) was used as a positive control.

### 2.17. Luciferase Reporter Assay

A total of 200,000 cells that were already transfected with siCtr or siSNAT1, respectively, were transfected with a 3xAP1pGL3 plasmid (RRID:Addgene_40342) [17], which was a gift from Alexander Dent, using Lipofectamine LTX Plus (Invitrogen, ThermoFisher Scientific). The empty vector pGL3basic (Promega; RRID:Addgene_212936) served as a control. The cells were lysed 24 h after plasmid transfection. Luciferase activity was quantified using the dual-luciferase reporter assay (Promega) following the manufacturer’s protocol. To normalize luminescence intensity, data were normalized to transfection efficiency by measuring renilla luciferase activity. For this purpose, the cells were co-transfected with 0.1 µg of the pRL-TK plasmid (Promega; RRID:Addgene_11313).

### 2.18. In Silico Modeling of Protein Structures

AlphaFold3 [18] (RRID:SCR_025885) modeling of the SNAT1-P62 complex was performed with standard settings. To investigate the effect of glutamine, four copies of (Gln)_4_, which is the smallest ligand that can be handled by AlphaFold3, were added to the modeling setup.

### 2.19. Statistical Analysis

All experiments in this study were conducted in at least three independent biological replicates. Data were depicted as the mean ± standard error of the mean (SEM) in the diagrams. The results were analyzed using the GraphPad Prism software (V 5.04; GraphPad Software, Inc., San Diego, CA, USA; RRID:SCR_002798). The following groups were compared using the Student’s unpaired *t*-test: siSNAT1 vs. siCtr; MeAIB vs. Ctr; V-9302 vs. Ctr; ZsGreen1-cMYC/pLVX-Puromycin vs. pLenti-C-GFP; HEK Ctr vs. HEK-SNAT1; and Ctr vs. Gln supplementation. Differences between groups were considered statistically significant at *p* > 0.05 (*: *p* < 0.05).

## 3. Results

### 3.1. SNAT1 Does Not Mediate Main Glutamine Influx, Despite Being Located in the Plasma Membrane

**Figure 1:**
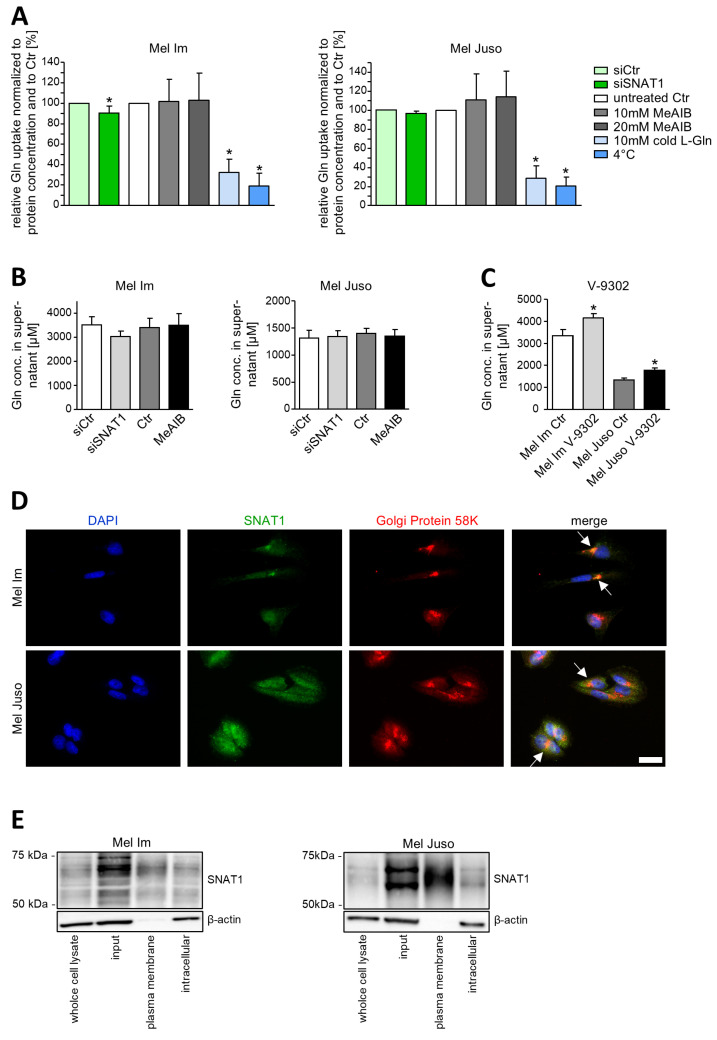
Glutamine uptake mediated by and localization of SNAT1 in melanoma cells. (**A**) Measurement of glutamine uptake into Mel Im and Mel Juso. Concentration of intracellular [3H]-glutamine was normalized to protein concentration and compared to the respective control (siCtr or untreated). Values represent the mean ± SEM of at least three independent experiments. (**B**) Analysis of extracellular glutamine concentration of Mel Im and Mel Juso after siSNAT1 transfection or application of the competitive SNAT-inhibitor MeAIB. Glutamine concentration was normalized to cell number. Values represent the mean ± SEM of three independent experiments. (**C**) Analysis of extracellular glutamine concentration of Mel Im and Mel Juso after treatment with ASCT2-inhibitor V-9302. Glutamine concentration was normalized to cell number. Values represent the mean ± SEM of four independent experiments. (**D**) Immunofluorescence staining of Mel Im and Mel Juso. Representative images of DAPI (blue), SNAT1 (green) and Golgi (red). Arrows indicate co-localization of SNAT1 and Golgi. Scale bar is 20 µm. (**E**) Exemplary images of surface biotinylation and subsequent Western Blot analysis of SNAT1 protein expression in Mel Im and Mel Juso. The treatment and respective control group were compared using the Student’s unpaired *t*-test. *p*-value < 0.05 was considered statistically significant (*). The uncropped western blot figures are presented in Supplementary Figure S5.

The main objective of this study was to investigate the molecular function of SNAT1. As this protein has been described as an amino acid influx symporter for sodium ions and neutral amino acids [8,9], of which glutamine is the most important one for cancer cells [5,19], we examined whether SNAT1 also functions as a glutamine transporter in melanoma cells. We measured the uptake of radioactively labeled glutamine into melanoma cells (Figure 1A). The decline in glutamine uptake when conducting the experiment at 4 °C instead of 37 °C is proof that glutamine uptake into melanoma cells is protein-mediated. Simultaneously incubating the cells with labeled and unlabeled (“cold”) glutamine at a higher concentration leads to reduced glutamine uptake, demonstrating that glutamine uptake can be inhibited. Surprisingly, in the cell line Mel Juso, siSNAT1 has no significant effect on glutamine uptake. In the cell line Mel Im, glutamine uptake is decreased only by 9.5% after knockdown of SNAT1. In addition, SNAT-inhibitor MeAIB, which inhibits SNAT1/2/4, has no significant influence on glutamine uptake, as well. We sequenced *SLC38A1* in several melanoma cell lines; however, no underlying mutation was observed that might explain why SNAT1 exerts no major glutamine transport function in melanoma cells (Supplementary Figure S1A). To confirm this interesting and new finding, we also analyzed the glutamine concentration of melanoma cells’ supernatant (Figure 1B). The results show that both siPool-mediated downregulation of SNAT1 expression and application of MeAIB have no significant effect on the glutamine concentration of cellular supernatant. Extracellular glutamine concentration reflects a combination of glutamine uptake, metabolism, and secretion. Therefore, this finding confirms that net uptake of glutamine from the medium is not primarily regulated by SNAT1 and is in line with our finding that SNAT1 is not the main glutamine transporter in melanoma. Moreover, as MeAIB is an unspecific inhibitor, our results might also hint at SNAT2 and SNAT4 not being the main glutamine influx transporters in melanoma, as well. To validate this, further experimental investigation is necessary in the future. When applying V-9302, which was designed as a specific and potent inhibitor for the glutamine transporter ASCT2 [20], glutamine concentration in the supernatant of melanoma cells is elevated significantly (Figure 1C). This possibly hints at ASCT2 being the loader for glutamine in melanoma, but it has to be investigated in more detail in a future study.

To address the question of whether SNAT1 is located in the plasma membrane of melanoma cells, we next examined the localization of this protein. Previous experiments conducting staining of melanoma tissue revealed that SNAT1 is located intracellularly in vivo [7]. To assess this in more detail, we conducted immunofluorescence staining, showing that SNAT1 is accumulated in intracellular membranes near the nucleus of melanoma cells (Figure 1D). Co-staining demonstrates that SNAT1 is located in the Golgi, where glycans are attached (Supplementary Figure S1B). Surface biotinylation of melanoma cells revealed that SNAT1 is not only located intracellularly, but also in the plasma membrane of melanoma cells (Figure 1E).

### 3.2. SNAT1 Does Not Regulate Downstream Targets of Amino Acid Starvation

**Figure 2:**
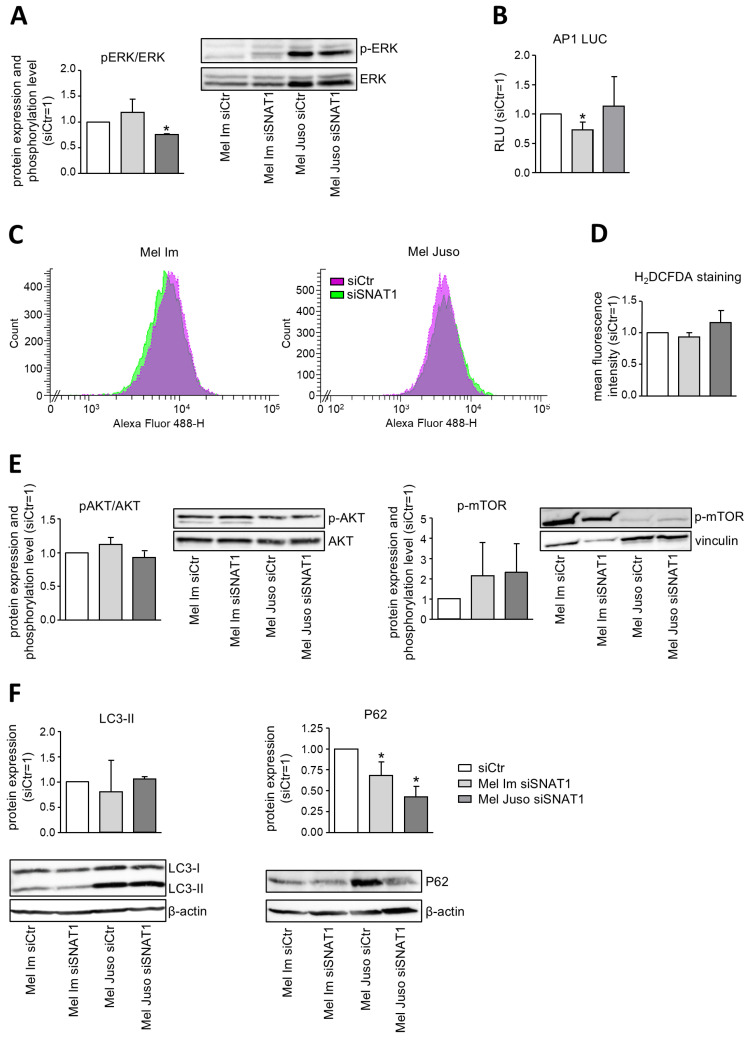
Analysis of glutamine-activated downstream effects and signaling pathways in melanoma. (**A**) Exemplary Western Blot image and densitometric analysis of pERK protein expression of Mel Im and Mel Juso. Protein expression was normalized to unphosphorylated ERK and compared to siCtr. Values represent the mean ± SEM of three independent experiments. (**B**) Luciferase assay of AP-1 promoter activity. Relative light units (RLU) were normalized to siCtr. Values represent the mean ± SEM of three independent experiments. (**C**) Exemplary cytometric measurement of DCF fluorescence intensity of Mel Im and Mel Juso after H_2_DCFDA staining. (**D**) Flow cytometric analysis of ROS formation using H_2_DCFDA staining by quantifying fluorescence intensity of DCF. Values represent the mean ± SEM of three independent experiments. (**E**) Western Blot and densitometric analysis of p-AKT and p-mTOR of Mel Im and Mel Juso. Protein expression was normalized to AKT or vinculin, respectively, and compared to siCtr. Values represent the mean ± SEM of three independent experiments. (**F**) Western Blot and densitometric analysis of autophagy markers P62 and LC3-II of Mel Im and Mel Juso. Protein expression was normalized to β-actin and compared to siCtr. Values represent the mean ± SEM of three independent experiments. The treatment and respective control group were compared using the Student’s unpaired *t*-test. *p*-value < 0.05 was considered statistically significant (*).

To clarify the molecular function of SNAT1 in melanoma, we first assessed whether SNAT1 might be involved in activation of the mitogen-activated protein kinase (MAPK) pathway, which is frequently and aberrantly activated in melanoma [21] (Figure 2A). Western Blot analysis demonstrated that the phosphorylation level of ERK is significantly reduced only in the cell line Mel Juso after knockdown of SNAT1 expression, whereas AP1-mediated promoter activity is diminished significantly only in the cell line Mel Im (Figure 2B). As AP-1 activity can be used as readout for JNK, ERK, or MAPK signaling [22], the data highlight that SNAT1 is not associated with consistent activation of these pathways in more than one melanoma cell line.

To further confirm our finding that SNAT1 is not implicated in the main glutamine influx of melanoma, we investigated the downstream effects of glutamine. Therefore, we assessed the possible formation of reactive oxygen species (ROS), as glutamine is an important precursor for the synthesis of glutathione [19] (Figure 2C,D). Downregulation of SNAT1 expression has no impact on fluorescence intensity of DCF, indicating that siSNAT1 does not induce the formation of ROS. As glutamine can activate mTOR [19], we also assessed whether SNAT1 is involved in activation of the AKT/mTOR signaling pathway (Figure 2E). We revealed in melanoma that SNAT1 is not connected to the activation of this pathway. In addition, we subsequently analyzed whether silencing of SNAT1 expression affects autophagy (Figure 2F and Supplementary Figure S2A). For this purpose, we assessed protein expression of autophagy markers P62 (*SQSTM1*) and LC3-II by performing Western Blot analysis. Knockdown of SNAT1 leads to significantly diminished protein expression of P62 but has no significant effect on LC3-II expression. This highlights that siSNAT1 does not induce autophagy; however, it hints at a strong regulation of P62. A previous publication revealed that P62 exerts an important function in melanoma progression by regulating mRNA stability of several pro-metastatic factors [23]. We confirmed that in melanoma, downregulation of SNAT1 expression leads to reduced expression of, e.g., FERMT2 mRNA (Supplementary Figure S2B). Taken together, these data support our finding that SNAT1 is not the primary glutamine transporter in melanoma but is involved in the regulation of intracellular signaling.

### 3.3. SNAT1 Activates P62/cMYC-Axis and Regulates cMYC Target Genes

**Figure 3:**
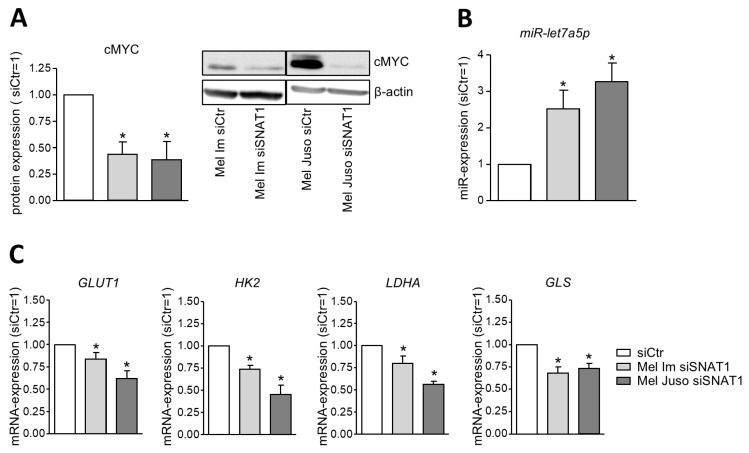
Activation of P62/cMYC axis by SNAT1. (**A**) Exemplary Western Blot image and densitometric analysis of cMYC protein expression of Mel Im and Mel Juso. Protein expression was normalized to β-actin and compared to siCtr. Values represent the mean ± SEM of four independent experiments. (**B**) Analysis of miR-let7a-5p expression by conducting qRT-PCR of Mel Im and Mel Juso. The expression level of miR-let7a-5p was normalized to U6 and compared to siCtr. Values represent the mean ± SEM of three independent experiments. (**C**) Analysis of mRNA expression of cMYC target genes GLUT1, HK2, LDHA, and GLS of Mel Im and Mel Juso was performed with qRT-PCR. The expression level was normalized to β-actin and compared to siCtr. Values represent the mean ± SEM of three independent experiments. The treatment and respective control group were compared using the Student’s unpaired *t*-test. *p*-value < 0.05 was considered statistically significant (*).

Interestingly, P62 protein expression is regulated by SNAT1. As a previous study demonstrated that P62 regulates cMYC via miR-let7a [24,25], we investigated cMYC and miR-let7a expression after siSNAT1 (Figure 3A,B). Silencing of SNAT1 leads to significantly reduced protein expression of cMYC and significantly elevated expression of miR-let7a. As cMYC is a transcription factor that regulates many aspects of tumor cell behavior, we explored its importance in SNAT1 signaling for melanoma cells in more detail. For this purpose, we determined mRNA expression of cMYC target genes that regulate metabolism (Figure 3C). Our qRT-PCR data demonstrate that knockdown of SNAT1 leads to significantly decreased mRNA expression of GLUT1, HK2, LDHA, and GLS, which were previously described as direct cMYC target genes [26]. This hints at a possible regulation of different metabolic pathways in melanoma siSNAT1 cells. Taken together, these data might indicate that in melanoma, SNAT1 regulates P62, which in turn regulates cMYC via miR-let7a.

### 3.4. SNAT1 Regulates Metabolism

**Figure 4:**
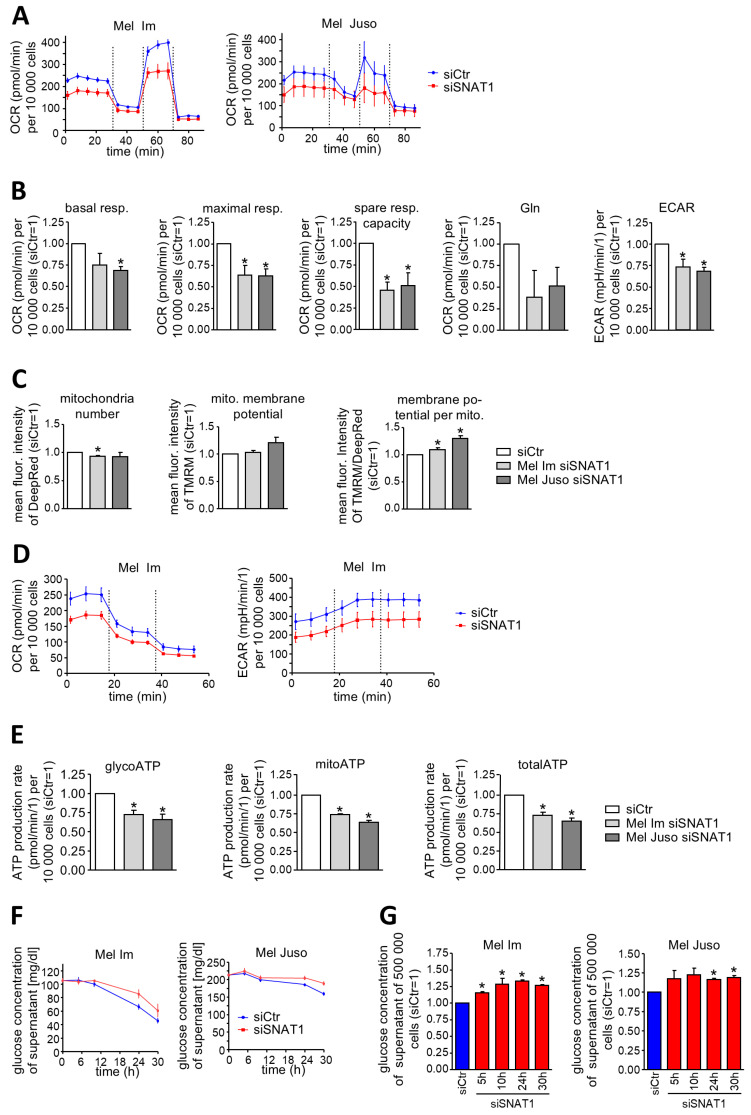
Regulation of mitochondrial respiration, ATP synthesis and glucose uptake by SNAT1 in melanoma. (**A**) Exemplary measurement of OCR of Mel Im and Mel Juso conducting Mito Stress Test, in which oligomycin, FCCP and rotenone/antimycin A are injected serially into the cells. (**B**) Calculation of basal respiration, maximal respiration, spare respiratory capacity, and ECAR of Mito Stress Test in Mel Im and Mel Juso. To assess glutamine metabolization, melanoma cells were starved for glutamine, glucose and pyruvate overnight, and OCR was measured after the addition of glutamine. OCR and ECAR were normalized to 10,000 cells and compared to siCtr. Values represent the mean ± SEM of at least three independent experiments. (**C**) Flow cytometric analysis of mitochondria number, mitochondrial membrane potential per cell and mitochondrial membrane potential per mitochondrion after staining of Mel Im and Mel Juso with TMRM and DeepRed. Values represent the mean ± SEM of three independent experiments. (**D**) Exemplary measurement of OCR and ECAR of Mel Im. During the ATP rate assay, Oligomycin and rotenone/antimycin A are injected serially into the cells. (**E**) Calculation of total ATP production and ATP synthesized via glycolysis and oxidative phosphorylation of ATP rate assay of Mel Im and Mel Juso. OCR and ECAR were normalized to 10,000 cells and compared to siCtr. Values represent the mean ± SEM of at least three independent experiments. (**F**) Exemplary time course of glucose concentration in the supernatant of Mel and Mel Juso. (**G**) Glucose concentration in the supernatant of Mel Im and Mel Juso was normalized to 500,000 cells and compared to siCtr. Values represent the mean ± SEM of three independent experiments. The treatment and respective control group were compared using the Student’s unpaired *t*-test. *p*-value < 0.05 was considered statistically significant (*).

To investigate in more detail the extent to which SNAT1 contributes to metabolic adaptation of melanoma cells, we performed the Mito Stress Test assay (Figure 4A,B). Quantification of OCR demonstrates that siSNAT1 significantly impairs basal respiration, maximal respiration and spare respiratory capacity in Mel Im and Mel Juso. In addition, siSNAT1 heavily impedes the metabolization of glutamine and significantly reduces ECAR in both melanoma cell lines we investigated. To analyze whether the reduced mitochondrial respiration is caused by alterations of the mitochondria, we stained melanoma cells with DeepRed and TMRM and subsequently analyzed them with flow cytometry (Figure 4C). DeepRed allows quantification of mitochondria, whereas TMRM enables the assessment of changes in the mitochondrial membrane potential. The number of mitochondria per cell is reduced slightly only in Mel Im. The mitochondrial membrane potential per cell is not altered, but the membrane potential per mitochondrion is elevated significantly in both cell lines. This might hint at a possibly existing compensation mechanism; however, it does not seem to be sufficient to balance out the impaired mitochondrial respiration. In addition, ATP synthesis is also impaired after knockdown of SNAT1 (Figure 4D,E). Conducting the ATP rate assay demonstrated that ATP synthesized via glycolysis (glycoATP) is reduced significantly after siSNAT1, as well as ATP generated via oxidative phosphorylation (mitoATP). Consequently, this leads to a significant decline in total ATP production.

To address the underlying cause of reduced ECAR, we analyzed glucose uptake of melanoma cells (Figure 4F,G). After silencing of SNAT1 expression, glucose concentration in the supernatant of melanoma cells is elevated significantly, suggesting that either glucose uptake or glycolysis is reduced after knockdown of SNAT1 expression.

**Figure 5:**
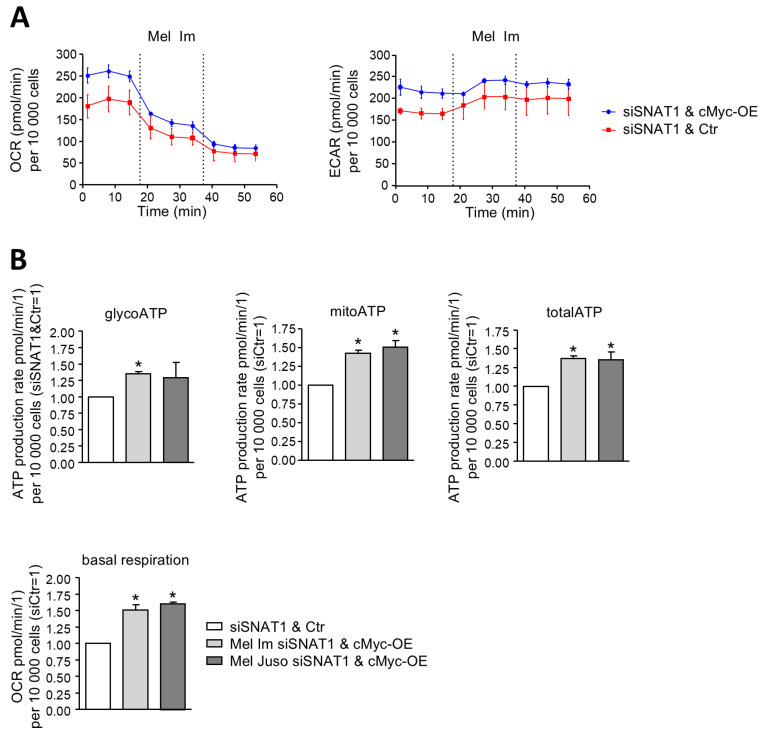
Validation of cMYC as a downstream effector of SNAT1 regarding ATP synthesis and mitochondrial respiration. (**A**) Exemplary measurement of OCR of Mel Im conducting the ATP rate assay. Melanoma cells were transfected with ZsGreen1-cMYC/pLVX-Puromycin or pLenti-C-GFP, respectively. (**B**) Calculation of ATP generated via glycolysis and oxidative phosphorylation, total ATP and basal respiration of Mel Im and Mel Juso. ATP production rate and OCR were normalized to 10,000 cells and compared to siCtr. Values represent the mean ± SEM of at least three independent experiments. The treatment and respective control group were compared using the Student’s unpaired *t*-test. *p*-value < 0.05 was considered statistically significant (*).

To further confirm the role of cMYC downstream of SNAT1 for metabolism of melanoma cells, we assessed whether we could rescue the metabolism of siSNAT1 cells by overexpressing cMYC (Figure 5A,B). For this purpose, we established overexpression of cMYC in siSNAT1 cells (Supplementary Figure S3A) using a ZsGreen1-cMYC/pLVX-Puromycin vector. Overexpression of cMYC in siSNAT1 cells significantly elevates ATP production during glycolysis and oxidative phosphorylation, leading to a significant increase in total ATP synthesis in melanoma cells (Figure 5B). In addition, overexpression of cMYC in siSNAT1 melanoma cells results in a significant increase in basal respiration (Figure 5B). The data also show that overexpression of cMYC after knockdown of SNAT1 expression leads to no significant changes in ATP synthesized via glycolysis and oxidative phosphorylation and of basal respiration compared to siCtr cells (Supplementary Figure S3B). This demonstrates that overexpression of cMYC leads to a rescue of these metabolic parameters back to siCtr levels. This highlights that elevated cMYC expression after silencing of SNAT1 expression attenuates impaired mitochondrial respiration and ATP biosynthesis of melanoma cells.

In conclusion, these results demonstrate that SNAT1 regulates mitochondrial respiration, glutaminolysis and glycolysis in melanoma cells, probably via its downstream effector cMYC.

### 3.5. SNAT1 Is a Transceptor for Gln and Captures P62

**Figure 6:**
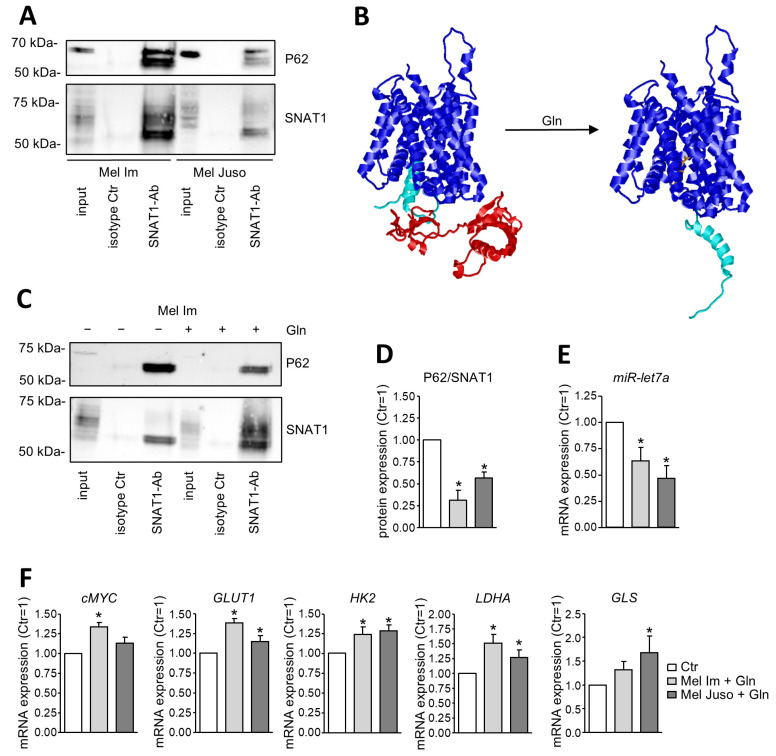
Glutamine-dependent interaction of SNAT1 and P62 in melanoma. (**A**) Exemplary image of CoIP and subsequent Western Blot analysis showing interaction of SNAT1 and P62 in untreated Mel Im and Mel Juso that were cultured under non-starved conditions in the presence of glutamine. (**B**) In silico model of the SNAT1-P62 complex (left structure). SNAT1 protein is depicted in blue, its N-terminal residues 35–70 in cyan; residues 1–170 of P62 are shown in red. In the presence of glutamine (right structure), SNAT1 residues 35–70 (cyan) adopt a different conformation, which might influence P62 binding. Glutamine is depicted in orange. (**C**) Exemplary Western Blot image of CoIP and subsequent Western Blot of Mel Im and Mel Juso after glutamine-starvation overnight. The following day, the treatment group was additionally incubated with glutamine-supplemented medium for 20 min before harvesting the cells. (**D**) Densitometric analysis of P62 protein binding to SNAT1 of Mel Im and Mel Juso. To quantify the binding affinity of P62 to SNAT1, P62 expression was normalized to SNAT1 protein expression. Values represent the mean ± of three independent experiments. (**E**) Quantification of miR-let7a-5p expression with qRT-PCR of Mel Im and Mel Juso. Expression level of miR-let7a-5p was normalized to U6 and compared to starved cells to which no glutamine was added. Values represent the mean ± SEM of three independent experiments. (**F**) Analysis of cMYC and its target genes’ mRNA expression in Mel Im and Mel Juso with qRT-PCR. The expression level was normalized to β-actin and compared to starved cells to which no glutamine was added. Values represent the mean ± SEM of at least three independent experiments. *p*-value of cMYC in Mel Juso is 0.0530 and of GLS in Mel Im is 0.0829. The treatment and respective control group were compared using the Student’s unpaired *t*-test. *p*-value < 0.05 was considered statistically significant (*).

To further focus on the molecular link between SNAT1 and P62, we performed Co-IP analyses demonstrating that SNAT1 and P62 are interacting in melanoma (Figure 6A) and HEK cells (Figure S4). To visualize a possible direct interaction of both proteins, we conducted in silico modeling using AlphaFold3 [18] (Figure 6B). Assuming that SNAT1 and P62 are direct interaction partners, the N-terminal half of P62 is predicted to interact with the intracellular part of SNAT1. Interestingly, binding of glutamine to SNAT1 is predicted to result in conformational changes, whereby the N-terminal residues 35–70 of SNAT1 are no longer inserted into the bundle of transmembrane helices, but protrude into the cytosol, which alters the shape of the predicted P62 binding site. This led us to hypothesize that binding of glutamine to SNAT1 and its associated rearrangement of the N-terminus have an impact on the binding affinity of P62 to SNAT1 and subsequent regulation of cMYC. To assess this, we starved melanoma cells overnight for glutamine and subsequently added glutamine the next day. Supplying melanoma cells with glutamine after starving significantly diminishes binding of P62 to SNAT1 (Figure 6C,D). Moreover, this leads to significantly reduced expression of miR-let7a (Figure 6E) and significantly elevated expression of cMYC and its target genes GLUT1, HK2, LDHA, and GLS (Figure 6F). Taken together, these data demonstrate that SNAT1 and P62 are interacting in melanoma and indicate that SNAT1-mediated activation of the P62/cMYC-axis is dependent on glutamine supply. This hints at SNAT1 functioning as a receptor for glutamine, thereby belonging to the category of transceptors.

## 4. Discussion

In this study, we newly revealed that SNAT1 is not the main glutamine transporter in melanoma, despite being located in the plasma membrane. Surprisingly, SNAT1 is still able to regulate the metabolism of melanoma cells. We discovered that SNAT1 interacts with P62 in a glutamine-dependent manner, likely as a transceptor. P62, in turn, regulates the expression of cMYC through miR-let7a, thereby activating transcription of cMYC target genes. Downregulation of SNAT1 expression results in regulation of mitochondrial respiration, ATP production, uptake of glucose, glycolysis, and glutaminolysis.

SNAT1 was previously described as a loader, being responsible for the main influx of neutral amino acids [10,27]. Amino acid transport mediated by SNAT1 was previously reported for X. laevis oocytes [9,10] and HRPE cells [8]. A study assessing the role of glutamine transporters in 143B and HeLa cells showed that when cells are cultured with sufficient glutamine, SNAT1 does not contribute significantly to glutamine uptake. However, under low glutamine concentrations, SNAT1 contribution to glutamine influx is elevated significantly, highlighting the crucial role of SNAT1 under amino acid depletion conditions [10]. Another study found that MeAIB has no significant impact on glutamine uptake of BxPC-3 and Panc03.27 cells, but did not investigate further the extent to which and under which conditions SNAT1 functions as a glutamine transporter in pancreatic cancer cells, therefore not drawing a conclusion about the transport activity of SNAT1 [28]. Surprisingly, our data show for the first time that SNAT1 is not involved in the main influx of glutamine into melanoma cells. In line with this, we previously published that silencing of SNAT1 expression in melanoma does not result in altered expression of glutamine transporters SNAT2, ASCT2, and LAT1 [7], demonstrating that knockdown of SNAT1 does not induce a compensatory upregulation of other glutamine influx transporters, especially of SNAT2, which has been described as a rescue transporter [10,27]. To further confirm that SNAT1 does not function as a main transporter for amino acids in melanoma, we also investigated downstream targets of glutamine transport. Glutamine deprivation commonly leads to elevated ROS levels [29]. However, we revealed that SNAT1 is not associated with ROS formation in melanoma. The AKT/mTOR signaling pathway can be activated by glutamine [19]. Other studies found that SNAT1 promotes activation of this pathway, proposing that its transported amino acids mediate AKT/mTOR activation [11,12,13]. In our study, SNAT1 does not activate this pathway. In line with this, we showed that SNAT1 does not induce autophagy in melanoma. These data support our finding that SNAT1 is not a primary glutamine transporter in melanoma, as depletion of amino acids is a typical trigger for autophagy induction in healthy and cancerous cells [30]. Several studies that investigated the importance of SNAT1 for other cancer entities did not assess its transport rate. Instead, functional effects were traced back to reduced glutamine uptake [11,12,15]. Therefore, it is unclear whether SNAT1, not being a main glutamine transporter, is specific for melanoma or not.

Our data regarding the application of V-9302 indicate that ASCT2 might be an important glutamine influx transporter in melanoma. V-9302 was designed as a specific inhibitor for ASCT2 and validated in vitro and in vivo [20,31]. In addition, knockdown of ASCT2 expression in melanoma cells reduces glutamine uptake [32]. However, the question of whether ASCT2 is the loader for glutamine in melanoma remains unclear, as another publication casts doubt on the specificity of this inhibitor and reports that SNAT2 and LAT1 are targets of V-9302, as well [27].

We were then curious to understand why SNAT1 has no significant transport function in melanoma. First, we verified that SNAT1 is located in the plasma membrane, which is in accordance with its predicted localization [8] and was experimentally shown in other studies [10,33]. Sequencing of the SLC38A1 gene revealed no mutation common to several melanoma cell lines. We confirmed that SNAT1 is glycosylated, which is in line with a previous study [33]. These posttranslational modifications are essential for the transport function of SNAT1 [33]. SNAT1-mediated transport of amino acids is coupled to sodium ions [8,9]. As we changed the cells’ medium 24 h before and again immediately before applying L-[3H]-glutamine, we can rule out deficiency of sodium ions as a possible cause of the missing transport function. The underlying cause of the transport deficiency of SNAT1 in melanoma remains unknown and requires further analysis, including crystal structure analysis.

Subsequently, we investigated the molecular function of SNAT1 in melanoma in more detail. Interestingly, we showed for the first time that SNAT1 regulates P62, miR-let7a and the oncogenic transcription factor cMYC as downstream effectors. The mechanism of P62 regulation by SNAT1 is not yet known. Possible molecular mechanisms could be the modulation of translation or protein stability. Investigating the molecular mechanism by which SNAT1 affects protein expression of P62 requires further investigation and constitutes a fruitful working program for the future. We demonstrated that these two proteins are interaction partners. P62 can regulate several metabolic pathways in cancer cells, which have been studied extensively, but the mechanisms behind this are not yet fully understood [34]. Previous studies showed that in some cancer entities cMYC is a downstream effector of P62, being regulated via miR-let7a [24,25]. P62 promotes mRNA stability of cMYC, resulting in increased cMYC protein expression [25]. The miR-let7a family is implicated in the development and progression of some cancer types [24,25]. In melanoma, expression of miR-let7a is downregulated, thereby contributing to a more invasive phenotype [35]. Taken together, these data indicate that SNAT1 activates cMYC via P62 and miR-let7a in melanoma. This finding is supported by the fact that a previous study found an association between SNAT1, miR-149-5p, and cMYC in colorectal cancer, but it was not assessed whether cMYC is regulated by SNAT1 or miR-149-5p [36]. A connection between SNAT1 and P62 was previously only drawn in the context of autophagy induction due to modulation of amino acid influx [13,37]. Interestingly, cMYC is also involved in the regulation of proliferation [38] and breaking of senescence [39], which are aspects of tumor cell behavior we detected after silencing of SNAT1 expression in melanoma cells [7]. While our study demonstrates significant changes at the mRNA level of cMYC target genes, we acknowledge that mRNA levels do not always perfectly correlate with protein abundance due to different post-translational regulation mechanisms. Future studies incorporating proteomic validation will be instrumental in further clarifying these dynamics.

We showed that SNAT1 knockdown diminishes glucose uptake and glycolysis. The connection of SNAT1 to glycolysis and acidification also implies that SNAT1 contributes to the Warburg effect [40]. This is in accordance with a previous study showing that silencing of SNAT1 expression reduces insulin-stimulated glucose consumption and lactate production. However, the regulation mechanism by which SNAT1 can regulate glucose uptake and lactate production was not investigated in this publication [41]. GLUT1, HK2, and LDHA are direct target genes of cMYC. Thereby, cMYC directly modulates glucose metabolism in cancer cells [26]. This is further supported by an in vivo study demonstrating that systemic P62 knockout mice were characterized by a generally reduced metabolic rate and glucose intolerance [42].

In addition, we also found that silencing SNAT1 results in decreased contribution of glutamine to mitochondrial respiration. As we already demonstrated that SNAT1 is not primarily involved in glutamine uptake, this implies that SNAT1 is rather associated directly with glutaminolysis. This is in line with our finding that SNAT1 modulates GLS expression, which is also a direct target gene of cMYC [26]. Elevated cMYC expression is considered a key driver of glutamine addiction in various cancer types by promoting a transcriptional program that shifts cellular bioenergetic reliance from glucose to glutamine, thereby increasing glutamine uptake [43].

In melanoma, SNAT1 is also a modulator of mitochondrial respiration and ATP synthesis. Alteration of mitochondrial membrane potential could hint at a possible compensation mechanism in melanoma. P62 deficiency is associated with a decreased mitochondrial membrane potential [44]. In mice, knockout of P62 impairs oxidative phosphorylation and ATP synthesis [45]. Interestingly, previous studies demonstrated that P62 regulates the expression of the majority of genes encoding proteins that constitute key components of the mitochondrial respiratory chain complexes. Thus, P62 promotes oxidative phosphorylation, thereby elevating mitochondrial respiration rate and oxygen consumption. However, this effect appears to be highly cell-type specific [44,46]. To the best of our knowledge, there are no data on P62 regulating proteins of the respiratory chain in melanoma. Reduced ATP synthesis also induces senescence [47], which is an effect we already reported in a previous study [7].

For the first time, we showed that SNAT1 and P62 are interacting, and that the strength of their interaction is modulated by extracellular glutamine, resulting in the regulation of miR-let7a, cMYC and its target genes. In the lysosomal transporter SNAT9 (*SLC38A9*) of zebrafish, the N-terminus is involved in conformational changes of this carrier protein. At a low luminal concentration of arginine, a substrate of SNAT9, the N-terminal tail of SNAT9 protrudes into the transmembrane pore of the protein. With an increasing concentration of arginine, the N-terminal tail is displaced from the substrate binding site and sticks out into the cytosol [48]. This is in line with our in silico modeling of SNAT1 conformation being dependent on glutamine supply and supports our data that even though SNAT1 does not transport glutamine in melanoma, it acts as a sensor for this amino acid. It is possible that, depending on the cell type, SNAT1 either acts as a transporter or sensor for glutamine, therefore constituting a transceptor. These are membrane proteins that fulfill two functions: solute transport and receptor-like signaling activities [49,50]. Many amino acid transporters, including members of the SLC38 family, were described as proteins that might function as transceptors independent of their transport function. Nevertheless, it is challenging to distinguish between these two functions, as the transported amino acids themselves can activate some signaling pathways [50]. A previous study demonstrated that expression and activity of cMYC is dependent on glutamine supply [51], but the mechanism behind this was not resolved. Our data newly provide a possible explanation for why cMYC expression is glutamine dependent.

## 5. Conclusions

In summary, we found that SNAT1 is not the main glutamine influx transporter in melanoma cells. Instead, SNAT1 and P62 are interaction partners in melanoma. SNAT1 activates the P62/cMYC-axis and target genes of cMYC in a glutamine-dependent manner, thereby modulating cellular metabolism. This indicates that SNAT1 constitutes a transceptor in melanoma. Our findings highlight the importance of SNAT1 for the progression of malignant melanoma, as deregulation of cellular energetics and metabolism is one of the new hallmarks of cancer [4].

## Figures and Tables

**Table 1:** Primers for qRT-PCR.

Primer	Forward Primer 5′-3′	Reverse Primer 5′-3′	Annealing Temperature	Measurement Temperature
*β-actin*	CTACGTCGCCCTGGACTTCGAGC	GATGGAGCCGCCGATCCACACGG	60 °C	
*cMYC*	GCTCCTGGCAAAAGGTCAGAGTCTGG	GGGGCTGGTGCATTTTCGGTTGTTGC	60 °C	76 °C
*GLS*	TGCTGGAAGCCTGCAAAGTA	CGGTTTGATTTTCCTTCCCGT	60 °C	76 °C
*GLUT1*	AACTCTTCAGCCAGGGTCCAC	CACAGTGAAGATGATGAAGAC	60 °C	76 °C
*HK2*	CAGCACAAAGCAGTCGGACC	GAGGCGCATGTGGTAGAGAT	60 °C	76 °C
*LDHA*	GGTTGGTGCTGTTGGCATGG	TGCCCCAGCCGTGATAATGA	60 °C	76 °C
*SQSTM1*/P62	CCGTGAAGGCCTACCTTCTG	TCCTCGTCACTGGAAAAGGC	60 °C	80 °C

## Data Availability

The data presented in this study are available upon request from the corresponding author.

## Supplementary Materials

The following supporting information can be downloaded at https://www.mdpi.com/article/10.3390/cancers18071068/s1, Figure S1: Sanger sequencing of *SLC38A1* and deglycosylation of SNAT1 protein in melanoma; Figure S2: mRNA expression of P62 and FERMT2 after knockdown of SNAT1 expression.; Figure S3: Establishment of cMYC overexpression in siSNAT1 melanoma cells; Figure S4: Interaction of SNAT1 and P62 in HEK cells. Figure S5: Uncropped Western Blots.

## Abbreviations

The following abbreviations are used in this manuscript:

AKT	Protein kinase B
miRNA	Micro-RNA
miRcDNA	miRNA-cDNA
Co-IP	Co-immunoprecipitation
MeAIB	α-(methylamino)isobutyric acid
H_2_DCFDA	2′,7′-dichlorodihydrofluorescein diacetate
FCCP	carbonyl cyanide-p-trifluoromethoxyphenylhydrazone
OCR	Oxygen consumption rate
ECAR	Extracellular acidification rate
TMRM	tetramethylrhodamin-methylester
SEM	Standard error of the mean
MAPK	Mitogen-activated protein kinase
ROS	Reactive oxygen species
RLU	Relative light units
glycoATP	ATP synthesized via glycolysis
mitoATP	ATP generated via oxidative phosphorylation
