# Supplementary material for: SNAT1 (SLC38A1) Is Not the Main Glutamine Transporter in Melanoma, but Controls Metabolism via Glutamine-Dependent Activation of P62 (SQSTM1)/cMYC-Axis
Source: Cancers (Basel). 2026 Mar 25;18(7):1068. doi: 10.3390/cancers18071068 (PMC13072269; doi:10.3390/cancers18071068)
Supplement: Supplementary file 1 [file cancers-18-01068-s001.zip › cancers-4188588-supplementary.pdf]

Supplemental Figures

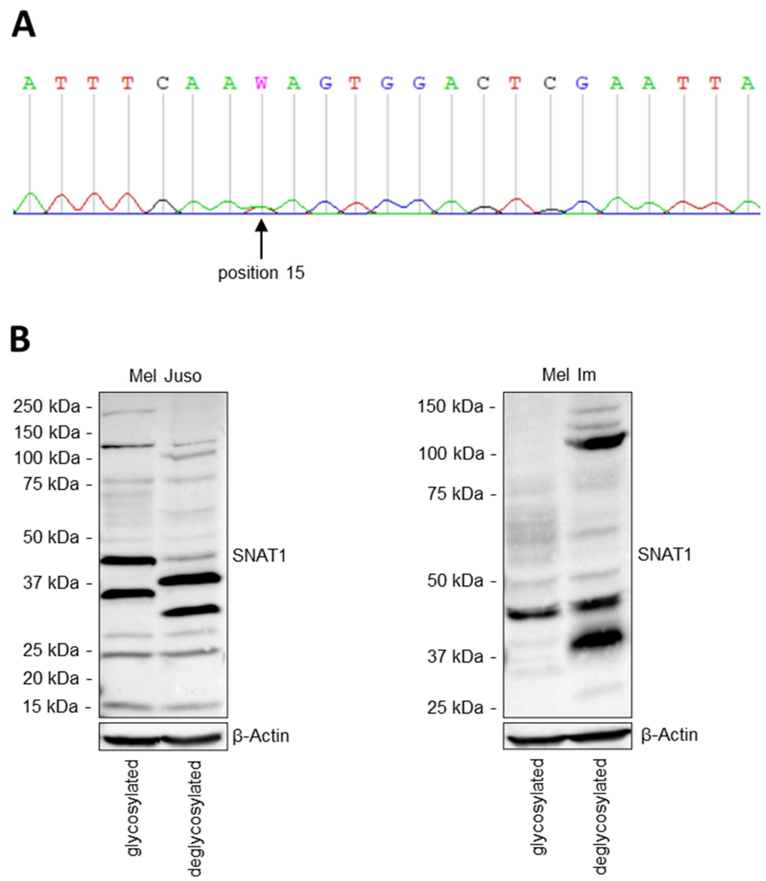

Supplemental Figure S1. Sanger sequencing of *SLC38A1* and deglycosylation of SNAT1 protein in melanoma. A) Sanger-Sequencing of *SLC38A1* (SNAT1) of cell line Mel Juso. Depicted is the beginning of the CDS of *SLC38A1*. At the marked position, heterozygous point mutation leads to overlap of thymine and adenine, resulting in base substitution. **B)** Representative Western Blots of Mel Im and Mel Juso after deglycosylation of protein lysates. Differences in band pattern show that native SNAT1 protein is glycosylated in melanoma cells.

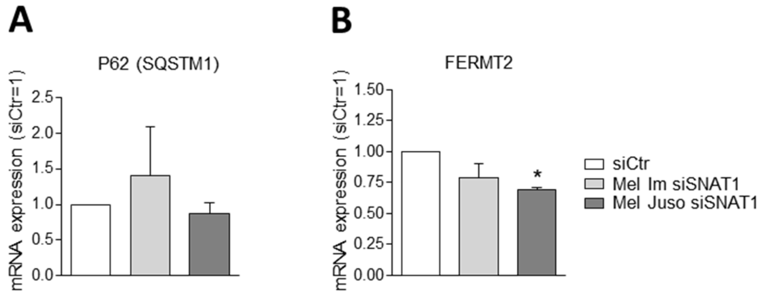

Supplemental Figure S2. mRNA expression of P62 and FERMT2 after knockdown of SNAT1 expression. A) Analysis of P62 (SQSTM1) mRNA expression using qRT-PCR of Mel Im and Mel Juso. Expression level was normalized to β-actin and compared to siCtr. Values represent the mean ± SEM of 4 independent experiments. **B)** Analysis of FERMT2 expression after SNAT1 knockdown. Analysis of FERMT2 mRNA expression with qRT-PCR of Mel Im and Mel Juso. Expression level was normalized to β-actin and compared to siCtr. Values represent the mean ± SEM of 3 independent experiments. Treatment and respective control group were compared using the student's unpaired t-test. p-value < 0.05 was considered statistically significant (*).

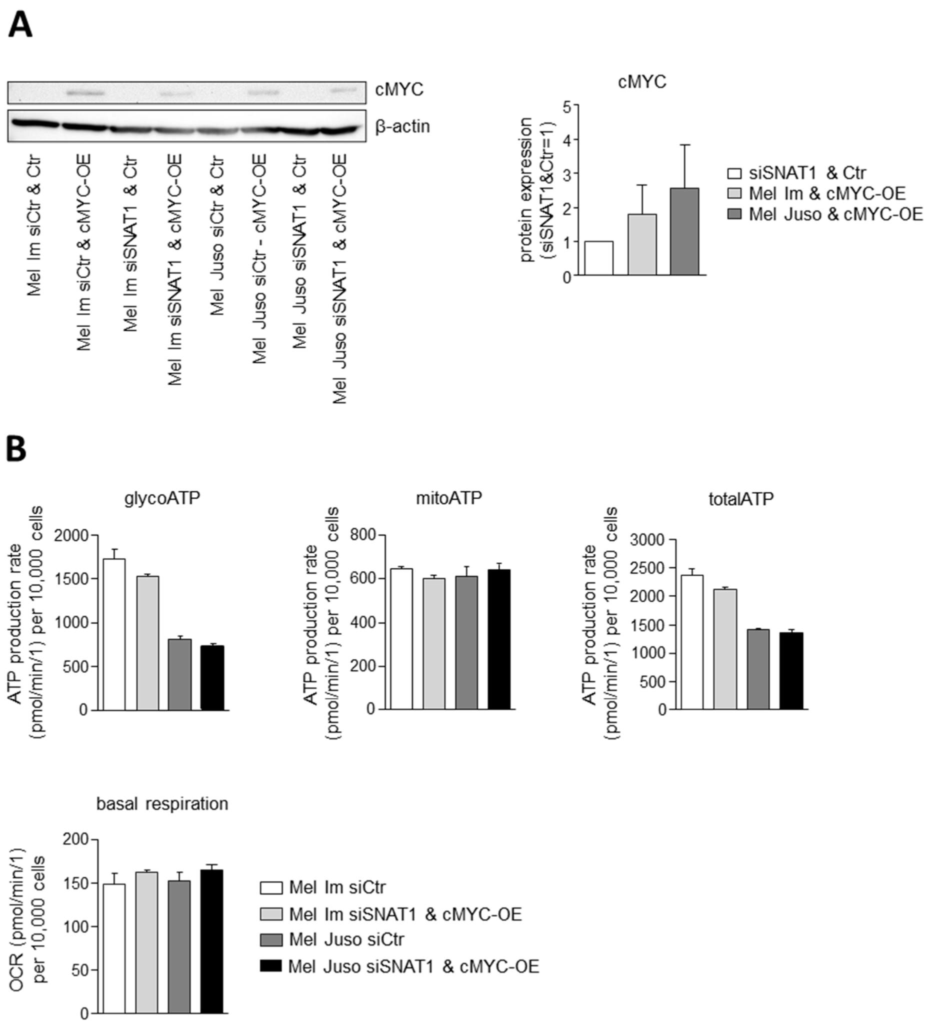

Supplemental Figure S3. Establishement of cMYC overexpression in siSNAT1 melanoma cells. A) Western Blot analysis and densitometric analysis of cMYC overexpression on the protein level of Mel Im and Mel Juso. Protein expression was normalized to β-actin and compared to siCtr. Values represent the mean ± SEM of 3 independent experiments. **B)** Calculation of ATP synthesized via glycolysis and oxidative phosphorylation, total ATP and basal respiration of Mel Im and Mel Juso. ATP production rate and OCR were normalized to 10,000 cells. Values represent the mean ± SEM of at least 3 independent experiments. Treatment and respective control group were compared using the student's unpaired t-test. p-value < 0.05 was considered statistically significant (*).

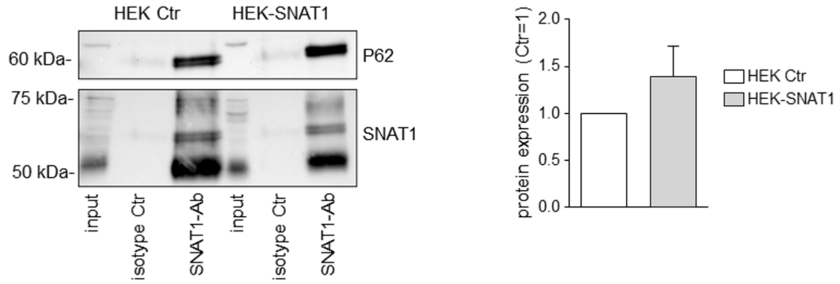

Supplemental Figure S4. Interaction of SNAT1 and P62 in HEK cells. Exemplary image of CoIP and subsequent Western Blot analysis showing interaction of SNAT1 and P62 in HEK with stable SNAT1 overexpression and HEK Ctr. Values represent the mean ± SEM of 3 independent experiments. Treatment and respective control group were compared using the student's unpaired t-test. p-value < 0.05 was considered statistically significant (*).

A

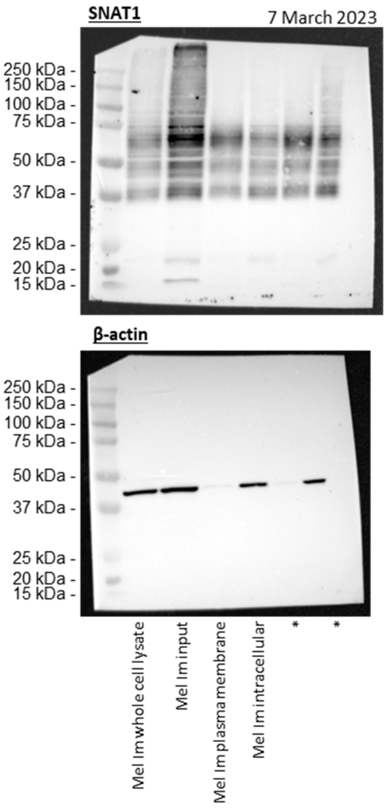
SNAT1
7 March 2023
250 kDa -
150 kDa -
100 kDa -
75 kDa -
50 kDa -
37 kDa -
25 kDa -
20 kDa -
15 kDa -
β-actin
250 kDa -
150 kDa -
100 kDa -
75 kDa -
50 kDa -
37 kDa -
25 kDa -
20 kDa -
15 kDa -
Mel Im whole cell lysate
Mel Im input
Mel Im plasma membrane
Mel Im intracellular
*
*

B

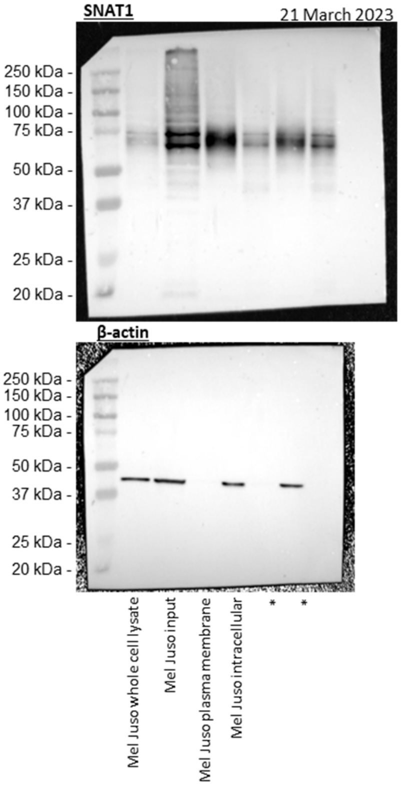
SNAT1
21 March 2023
250 kDa -
150 kDa -
100 kDa -
75 kDa -
50 kDa -
37 kDa -
25 kDa -
20 kDa -
β-actin
250 kDa -
150 kDa -
100 kDa -
75 kDa -
50 kDa -
37 kDa -
25 kDa -
20 kDa -
Mel Juso whole cell lysate
Mel Juso input
Mel Juso plasma membrane
Mel Juso intracellular
*
*

C

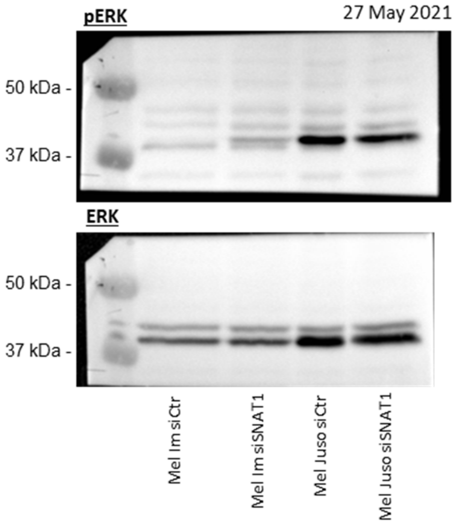
pERK
27 May 2021
50 kDa -
37 kDa -
ERK
50 kDa -
37 kDa -
Mel Im siCtr
Mel Im siSNAT1
Mel Juso siCtr
Mel Juso siSNAT1

D

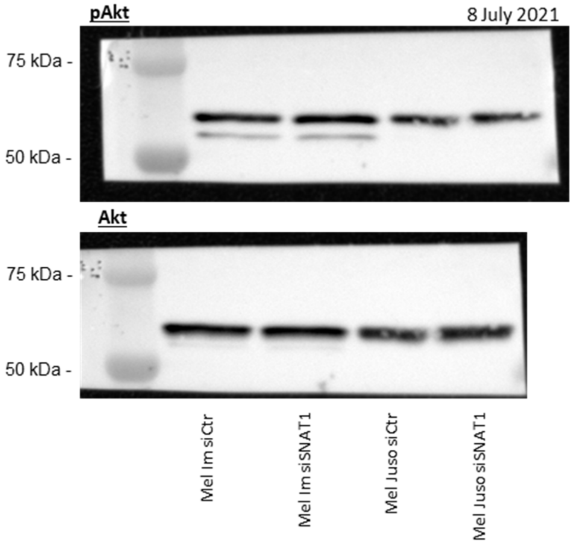
pAkt
8 July 2021
75 kDa -
50 kDa -
Akt
75 kDa -
50 kDa -
Mel Im siCtr
Mel Im siSNAT1
Mel Juso siCtr
Mel Juso siSNAT1

E

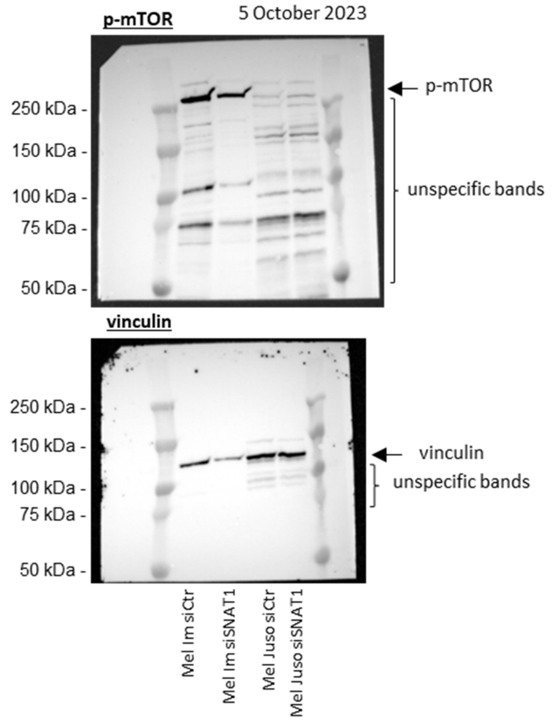
p-mTOR
5 October 2023
250 kDa -
150 kDa -
100 kDa -
75 kDa -
50 kDa -
p-mTOR
unspecific bands
vinculin
250 kDa -
150 kDa -
100 kDa -
75 kDa -
50 kDa -
vinculin
unspecific bands
Mel Im siCtr
Mel Im siSNAT1
Mel Juso siCtr
Mel Juso siSNAT1

F

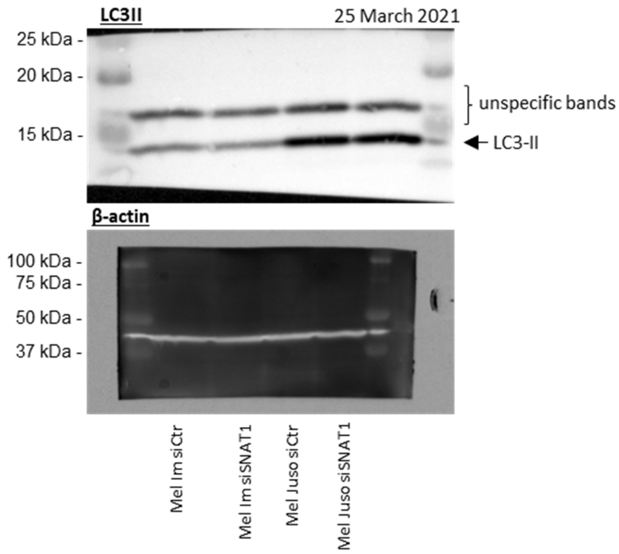
LC3II
25 March 2021
25 kDa -
20 kDa -
15 kDa -
unspecific bands
LC3-II
β-actin
100 kDa -
75 kDa -
50 kDa -
37 kDa -
Mel Im siCtr
Mel Im siSNAT1
Mel Juso siCtr
Mel Juso siSNAT1

G

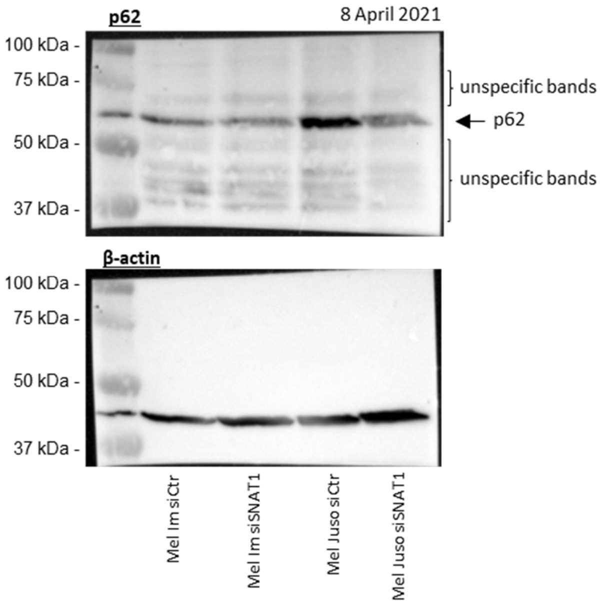
p62
8 April 2021
100 kDa -
75 kDa -
50 kDa -
37 kDa -
unspecific bands
p62
unspecific bands
β-actin
100 kDa -
75 kDa -
50 kDa -
37 kDa -
Mel Im siCtr
Mel Im siSNAT1
Mel Juso siCtr
Mel Juso siSNAT1

H

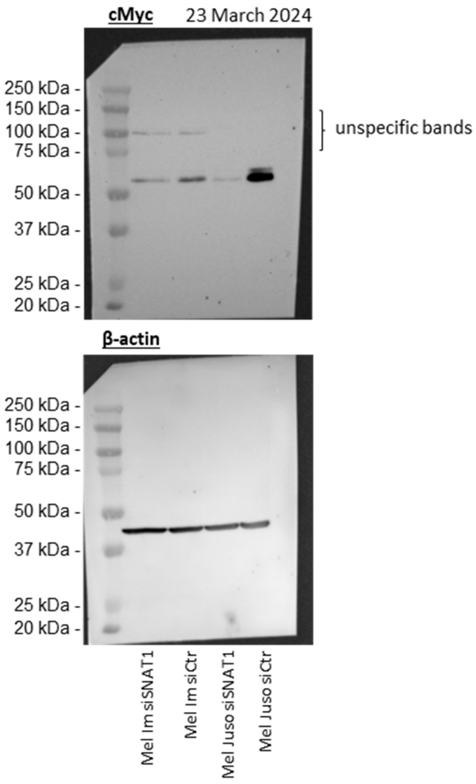
cMyc
23 March 2024
250 kDa -
150 kDa -
100 kDa -
75 kDa -
50 kDa -
37 kDa -
25 kDa -
20 kDa -
unspecific bands
β-actin
250 kDa -
150 kDa -
100 kDa -
75 kDa -
50 kDa -
37 kDa -
25 kDa -
20 kDa -
Mel Im siSNAT1
Mel Im siCtr
Mel Juso siSNAT1
Mel Juso siCtr

I

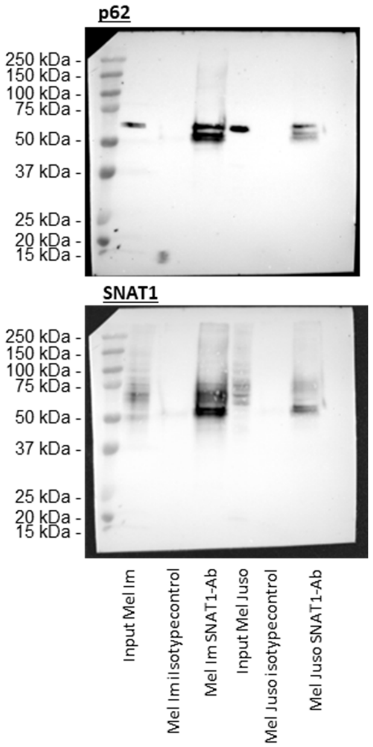
p62
250 kDa -
150 kDa -
100 kDa -
75 kDa -
50 kDa -
37 kDa -
25 kDa -
20 kDa -
15 kDa -
SNAT1
250 kDa -
150 kDa -
100 kDa -
75 kDa -
50 kDa -
37 kDa -
25 kDa -
20 kDa -
15 kDa -
Input Mel Im
Mel Im iIsotypecontrol
Mel Im SNAT1-Ab
Input Mel Juso
Mel Juso isotypecontrol
Mel Juso SNAT1-Ab

J

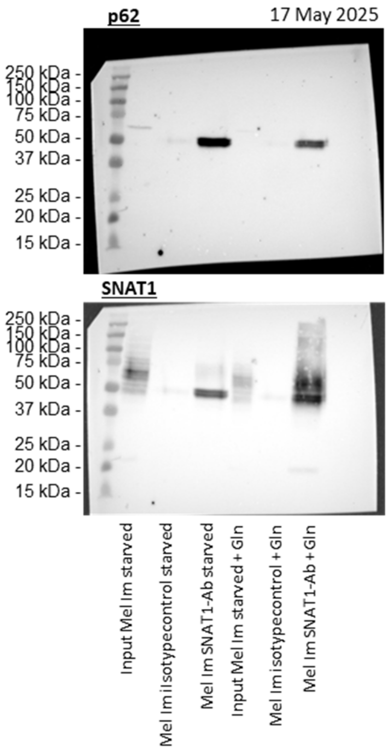
p62
17 May 2025
250 kDa -
150 kDa -
100 kDa -
75 kDa -
50 kDa -
37 kDa -
25 kDa -
20 kDa -
15 kDa -
SNAT1
250 kDa -
150 kDa -
100 kDa -
75 kDa -
50 kDa -
37 kDa -
25 kDa -
20 kDa -
15 kDa -
Input Mel Im starved
Mel Im iIsotypecontrol starved
Mel Im SNAT1-Ab starved
Input Mel Im starved + Gln
Mel Im isotypecontrol + Gln
Mel Im SNAT1-Ab + Gln

K

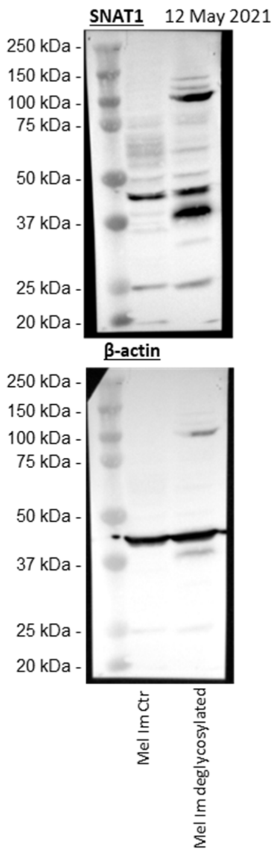
SNAT1
12 May 2021
250 kDa -
150 kDa -
100 kDa -
75 kDa -
50 kDa -
37 kDa -
25 kDa -
20 kDa -
β-actin
250 kDa -
150 kDa -
100 kDa -
75 kDa -
50 kDa -
37 kDa -
25 kDa -
20 kDa -
Mel Im Ctr
Mel Im deglycosylated

L

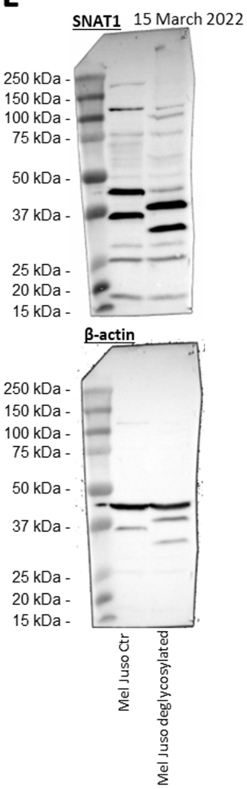
SNAT1
15 March 2022
250 kDa -
150 kDa -
100 kDa -
75 kDa -
50 kDa -
37 kDa -
25 kDa -
20 kDa -
15 kDa -
β-actin
250 kDa -
150 kDa -
100 kDa -
75 kDa -
50 kDa -
37 kDa -
25 kDa -
20 kDa -
15 kDa -
Mel Juso Ctr
Mel Juso deglycosylated

M

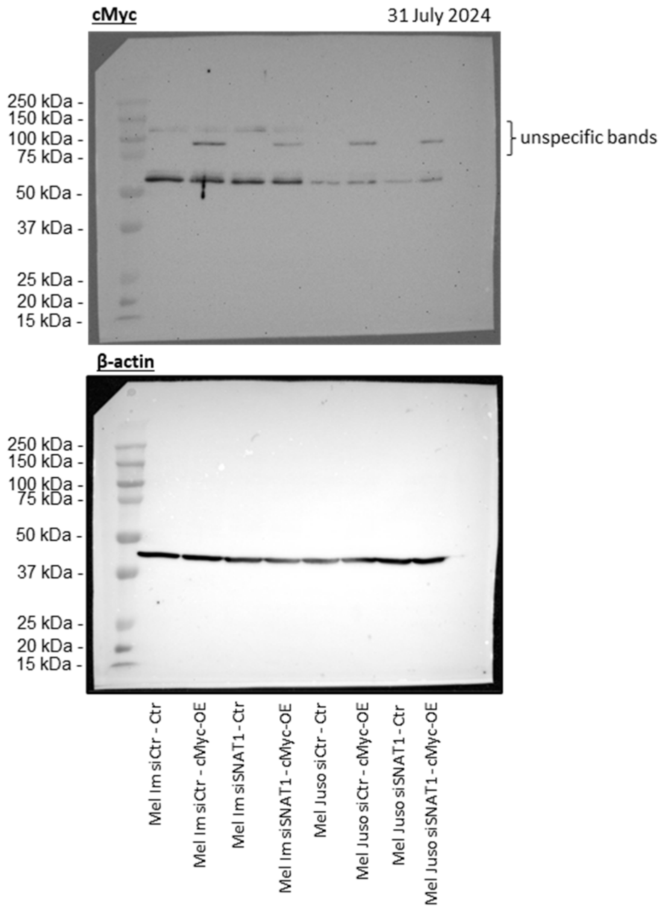
cMyc
31 July 2024
250 kDa -
150 kDa -
100 kDa -
75 kDa -
50 kDa -
37 kDa -
25 kDa -
20 kDa -
15 kDa -
unspecific bands
β-actin
250 kDa -
150 kDa -
100 kDa -
75 kDa -
50 kDa -
37 kDa -
25 kDa -
20 kDa -
15 kDa -
Mel Im siCtr - Ctr
Mel Im siCtr - cMyc-OE
Mel Im siSNAT1 - Ctr
Mel Im siSNAT1 - cMyc-OE
Mel Juso siCtr - Ctr
Mel Juso siCtr - cMyc-OE
Mel Juso siSNAT1 - Ctr
Mel Juso siSNAT1 - cMyc-OE

N

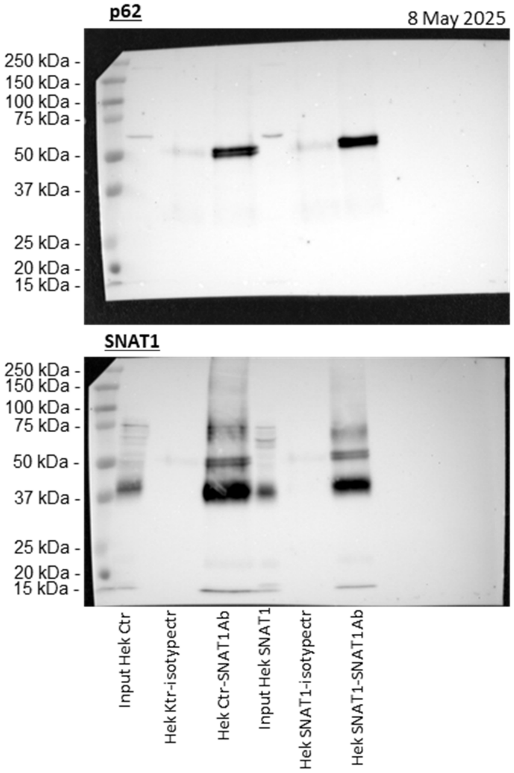

Supplementary Figure S5. Uncropped Western Blots. A-B) Western Blot analysis of SNAT1 and β-actin protein level of indicated melanoma cell lines. Associated with Figure 1E. **C)** Western Blot analysis of pERK and ERK protein level of indicated melanoma cell lines. Associated with Figure 2A. **D)** Western Blot analysis of pAkt and Akt protein level of indicated melanoma cell lines. Associated with Figure 2E. **E)** Western Blot analysis of p-mTOR and vinculin protein level of indicated melanoma cell lines. Associated with Figure 2E. **F)** Western Blot analysis of LC3II and β-actin protein level of indicated melanoma cell lines. Associated with Figure 2F. **G)** Western Blot analysis of p62 and β-actin protein level of indicated melanoma cell lines. Associated with Figure 2F. **H)** Western Blot analysis of cMyc and β-actin protein level of indicated melanoma cell lines. Associated with Figure 3A. **I)** Western Blot analysis of p62 and SNAT1 protein level of indicated melanoma cell lines. Associated with Figure 6A. **J)** Western Blot analysis of p62 and SNAT1 protein level of indicated melanoma cell lines. Associated with Figure 6C. **K-L)** Western Blot analysis of SNAT1 and β-actin protein level of indicated melanoma cell lines. Associated with Figure S1B. **M)** Western Blot analysis of cMyc and β-actin protein level of indicated melanoma cell lines. Associated with Figure S3A. **N)** Western Blot analysis of p62 and SNAT1 protein level of indicated melanoma cell lines. Associated with Figure S4. Lanes labeled with an asterisk (*) contain samples that are unrelevant for this study. Dates indicate time of development of the blots.
